# Realist evaluation of Schwartz rounds® for enhancing the delivery of compassionate healthcare: understanding how they work, for whom, and in what contexts

**DOI:** 10.1186/s12913-021-06483-4

**Published:** 2021-07-18

**Authors:** J. Maben, C. Taylor, E. Reynolds, I. McCarthy, M. Leamy

**Affiliations:** 1 School of Health Sciences, Faculty of Health and Medical Sciences, University of Surrey, Kate Granger Building, Priestley Road, Surrey Research Park, Guildford, GU2 7YH UK; 2 CAMERA Research Group, Plymouth Institute of Health and Care Research, Faculty of Health, Plymouth University, Drake Circus, Plymouth, Devon, PL4 8AA UK; 3 Aston Business School, Aston University, Aston Triangle, Birmingham, B4 7ET UK; 4 Care of Long term Conditions Research Division, Florence Nightingale Faculty of Nursing, Midwifery & Palliative Care, King’s College London, James Clerk Maxwell building, Waterloo Road, London, SE1 8WA UK

**Keywords:** Schwartz rounds, Healthcare professionals, Emotional impact, Staff well-being, Reflection, Compassion, Compassionate care, Empathy; culture change, Staff experiences

## Abstract

**Background:**

Healthcare work is known to be stressful and challenging, and there are recognised links between the psychological health of staff and high-quality patient care. Schwartz Center Rounds® (Rounds) were developed to support healthcare staff to re-connect with their values through peer reflection, and to promote more compassionate patient care. Research to date has focussed on self-report surveys that measure satisfaction with Rounds but provide little analysis of how Rounds ‘work’ to produce their reported outcomes, how differing contexts may impact on this, nor make explicit the underlying theories in the conceptualisation and implementation of Rounds.

**Methods:**

Realist evaluation methods aimed to identify how Rounds work, for whom and in what contexts to deliver outcomes. We interviewed 97 key informants: mentors, facilitators, panellists and steering group members, using framework analysis to organise and analyse our data using realist logic. We identified mechanisms by which Rounds lead to outcomes, and contextual factors that impacted on this relationship, using formal theory to explain these findings.

**Results:**

Four stages of Rounds were identified. We describe how, why and for whom Schwartz Rounds work through the relationships between nine partial programme theories. These include: trust safety and containment; group interaction; counter-cultural/3rd space for staff; self-disclosure; story-telling; role modelling vulnerability; contextualising patients and staff; shining a spotlight on hidden stories and roles; and reflection and resonance. There was variability in the way Rounds were run across organisations. Attendance for some staff was difficult. Rounds is likely to be a ‘slow intervention’ the impact of which develops over time. We identified the conditions needed for Rounds to work optimally. These contextual factors influence the intensity and therefore degree to which the key ingredients of Rounds (mechanisms) are activated along a continuum, to produce outcomes. Outcomes included: greater tolerance, empathy and compassion for self and others; increased honesty, openness, and resilience; improved teamwork and organisational change.

**Conclusions:**

Where optimally implemented, Rounds provide staff with a safe, reflective and confidential space to talk and support one another, the consequences of which include increased empathy and compassion for colleagues and patients, and positive changes to practice.

## Background

Staff shortages and the increase in complexity in healthcare provision have had a negative impact on healthcare staff who may experience excessive levels of psychological distress, face increasing demand, scrutiny, and regulation, and are subject to economic cutbacks [1, 2]. Stress is an antecedent of various distressing psychological states such as loss of ideals and burnout, which can reduce compassion and empathy [3–5]. Previous research has clearly demonstrated the relationships between staff wellbeing and patient care [6–8]. Schwartz Center Rounds® (Rounds) were created to help healthcare staff provide compassionate care by encouraging staff to reflect on their work and rediscover what initially attracted them to healthcare work [9, 10].

Rounds were inspired by the experiences of a Boston healthcare lawyer, Kenneth Schwartz, whose experiences as a patient when terminally ill with lung cancer, led him to write that ‘small acts of kindness make the unbearable bearable’ [11]. Schwartz specifically noted the value of healthcare workers engaging with him as a person and displaying empathy. Before he died in 1995, Schwartz established a not-for-profit organisation called the Schwartz Center for Compassionate Care (SCCC) in order to develop and later implement Rounds across the USA. In 2009 Rounds were brought to the UK by the Point of Care Foundation (PoCF) who held a licence to run Rounds with the SCCC. Over 420 healthcare organisations in the USA, and over 200 in the UK and Ireland, hold licences to run Rounds which are also beginning to be implemented in Australia and other countries.

In the UK, Rounds take the form of usually monthly group meetings open to everyone in the organisation, both clinical and non-clinical staff. They provide staff with structured time and a confidential, safe space to talk about and reflect upon the social, ethical and emotional challenges of looking after patients and their families in a protected safe space.

**Fig. 1:**
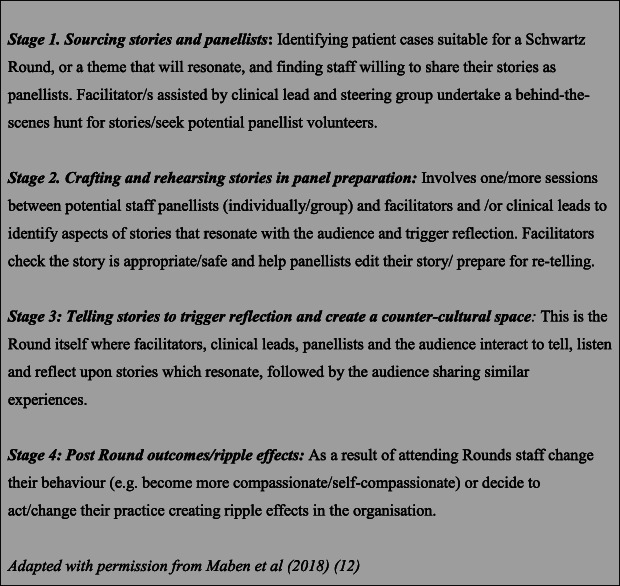
Description of Four Round Stages

Our understanding of Rounds comes from the team’s wider body of work [12] where we, uniquely, identified four stages of a Round (Fig. 1). Two occurred before the Rounds itself (Stages 1 and 2 sourcing stories and panellists and crafting and rehearsing stories in panel preparation); and one after the Round (Post Round outcomes/ripple effects) with stage 3 being the Round itself.

The fourth stage of a Round, or a succession of Rounds, influences the initial stages of subsequent Rounds, producing a repository of knowledge and understanding (resources and context) which enriches subsequent Rounds. In our comparison of new and established sites, the pivotal role of this cumulative effect became clear; it shaped the confidence and trust which emerged between audience and facilitator.

The focus in Rounds is on the impact on staff of providing care, not solving problems or focusing upon the clinical aspects of patient care [12]. There is often a temptation to problem-solve or examine clinical aspects of care within the Round, but the focus is brought back to the reflections of staff on the experience of caring for patients and their families [12]. Each Round lasts for 1 h and starts with a pre-prepared presentation by a multidisciplinary panel of up to four staff members of a patient case by the team who cared for the patient, or a set of different stories based around a common theme. The panellists each describe the impact on them of the difficult, demanding or satisfying aspects of the situation and the topic is then opened to the audience for group reflection and discussion. Two trained facilitators – usually a senior clinician and psychosocial practitioner – are present to support and steer the discussion of themes as they emerge, allowing time for the audience to comment and / or reflect on similar experiences they may have had if they wish. A steering group helps support Rounds and a senior doctor/clinician is required to champion Rounds within the organisation. Rounds are voluntary to attend, and staff are free to attend as many or few as they wish. Rounds are usually held at lunchtime with food provided. Rounds are designed to support staff to remain engaged with their work, and compassionate towards patients, their families, and colleagues, to improve the wellbeing of staff, effectiveness of communication and engagement, and, ultimately the care of patients [9, 10, 13].

There are few evaluations of the efficacy of Rounds but more are taking place as they become more widespread. Evidence from the USA and UK suggests that attending Rounds is associated with improved wellbeing and relationships with colleagues, and more empathic and compassionate patient care [12]. Evidence shows Rounds to be highly valued by attendees and most studies reported positive impact on ‘self’ (e.g. improved wellbeing, improved ability to cope with emotional difficulties at work, self-reflection/validation of experiences), [9, 10, 14–19] and impact on patients (increased compassion, empathy) [9, 10, 14, 15, 17, 20], and colleagues (improved teamwork, compassion/empathy), [9, 10, 14–16, 18–20]. However the evidence base is of low to moderate quality as the studies generally used cross-sectional designs and self-report views/satisfaction with Rounds. They give scant analysis or insight into what occurs during the Round, including how or why Rounds work to produce outcomes.

This article presents data from the first large-scale evaluation of Rounds in the UK. Using realist evaluation methodology [21] it also aimed to identify how an intervention (in this case Rounds) works, for whom and in which contexts to deliver outcomes, and thereby aimed to make explicit what is happening in a Round and across the four stages of Rounds identified above. It reports salient findings about the ways in which Rounds work, with a focus on the optimum components that maximise their effectiveness.

## Methods

This paper reports part of a wider National Institute of Health Research (NIHR) study and whilst this paper reports substantial methodological detail, further details of the methods of the wider study can be found in the overall study report [12]. This study used a realist evaluation methodology, a theory-driven approach which aims to develop and refine an evidence-based theory to explain how, why and for whom an intervention works [22]. Steps included:

1. identifying an initial programme theory (IPT) (which we did by reviewing the literature; interviewing those who designed Schwartz Rounds in the USA and those who brought them to the UK; and reviewing documents – see below);
2. carrying out and analysing realist interviews with Rounds key stakeholders to refine IPT and to develop partial evidenced-informed programme theories;
3. The use and integration of formal theory to build our partial programme theories and interpret our findings (see Fig. 5 below for formal theories used in this paper).
4. carrying out and retroductively analysing focus group discussions with key stakeholders to test evidence-informed programme theories to compare how Rounds work, for whom and in what context and compare this to our initial programme theory to develop our model of how Rounds work for whom in what contexts (Fig. 9 model).

**Fig. 2:**
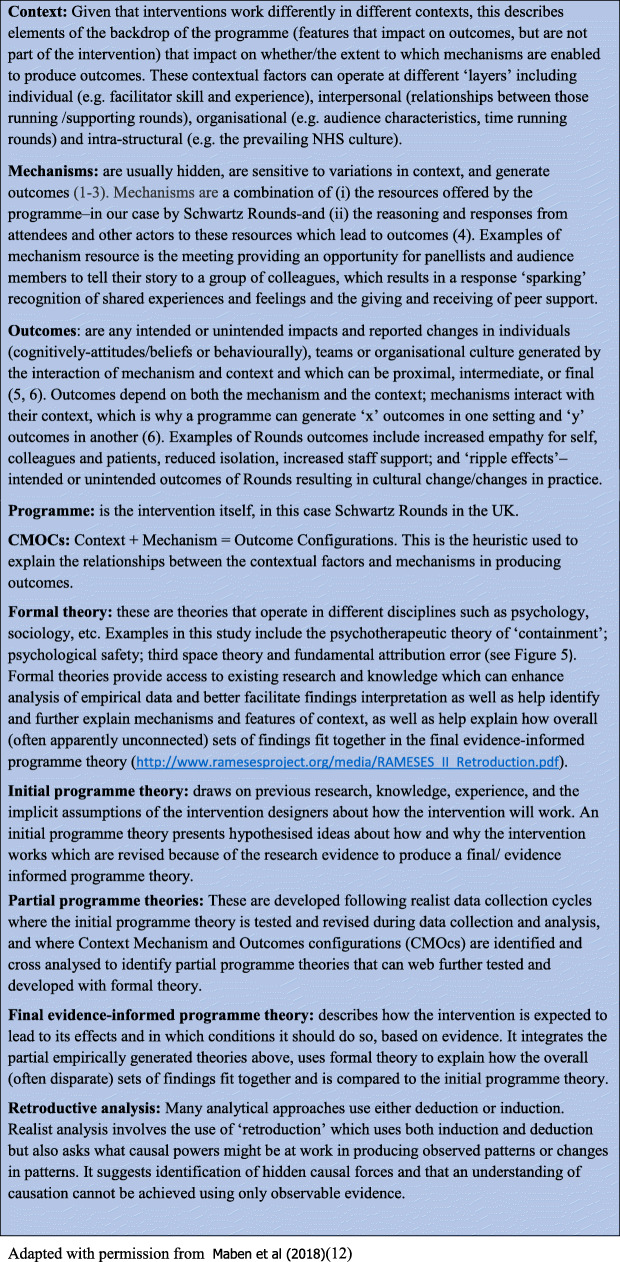
Definitions of realist evaluation terms

Several key principles guided this approach and these steps, firstly that the same intervention in different contexts with different participants will produce different outcomes (people create the outcomes/change, not the intervention); and second, that interventions (in this case Rounds) offer resources that people can choose to respond to or not. This resource-response action is called a mechanism. Mechanisms are often hidden (require depth of analysis to surface them) and may work differently in different contexts [21, 23, 24]. The way that context influences mechanisms, and thereby outcomes, is articulated using a heuristic called the ‘context + mechanism = outcome’ configuration [21, 22]. Figure 2 explains further some of the key Realist terms.

The overarching aim in a realist evaluation is thus to understand the complex relationship between mechanisms and the effect that context has on their operationalisation and outcome [21, 25].

All initiatives will (implicitly or explicitly) have a programme theory or theories [23] about how they are expected to cause their intended outcomes. When a programme like Schwartz Rounds is implemented, it is implicitly testing a theory about what ‘might cause change’, even though that theory may not be overt [26].

### Initial Programme theory development

A key aim of our realist evaluation was to uncover and make explicit the explanations for how, why and for whom Schwartz Rounds work. Specific data used to develop our initial programme theory included our:

(i) initial review of the literature to define Rounds [27] and identify potential mechanisms by which they may work [12](e.g. the resource-response relationships referred to earlier).
(ii) interviews with programme architects in the USA (*n* = 2) and UK Schwartz initial implementers (n = 2), analysed to identify themes and core underlying guiding principles of Rounds [12, 28]
(iii) review of programme documentation including SCCC, Boston and PoCF websites and contracts (prior to implementing Rounds organisations sign a contract with the SCCC in USA / PoCF in UK) (see Leamy et al. (2019) [28].

**Fig. 3:**
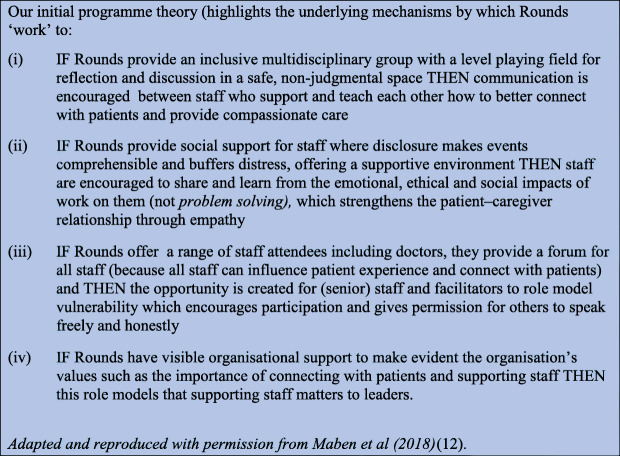
*Initial programme theory*

Following analysis of interview data and programme documents using thematic framework analysis [29], extensive iteration and team discussion we finalised our initial programme theories about how, for whom, and under what circumstances Rounds work [12] (Fig. 3).

### Developing partial and final evidence-informed programme theory

Our realist evaluation sought to refine and test this initial programme theory (Fig. 3) through further data collection cycles (see below) to produce (i) first a set of partial programme theories with supporting CMOCs drawing on formal theory to interpret our findings in the context of existing research and knowledge about a topic and then (ii) finally integrating these partial theories to produce a revised evidence-informed programme theory.

### Sample and data collection

With ethics approval granted by (King’s College London University ethics committee) and the National Research Ethics Service Committee London**-**South East (REC reference: 15/LO/0053) we interviewed a purposive sample of 48 facilitators and clinical leads in 45 sites running Rounds in 2015 by telephone. These participants were approached by email, and invited to participate. A small number did not respond to email invitations and so did not participate. The interview topic guide is published in our study report [12]. Nine organisational case studies (acute/mental health/community Trusts and hospices in England) were also purposively sampled nationally (UK) to provide maximum variation, (size of institution, length of time running Rounds; established and new Rounds and early and late adopters). Data collected in these case studies included interviews with a purposive sample of clinical leads, facilitators, panellists, and members of steering groups, audiences, organisation Boards and non-attenders (*n* = 177). These participants were approached by email or face-to-face. A small number were not able to participate due to clinical commitments, or did not respond to email invitations. The interview guide for these interviews and topics covered in the focus groups is published in our study report [12]. In total therefore we had (*n* = 225) interviews of which we analysed *n* = 97 key informant interviews (primarily mentors, facilitators, clinical leads, although some were also panellists and steering group members) for the realist analysis reported in this article. These interviews were most helpful in addressing our aims because key informant participants had thought most about how Rounds ‘worked’ and changed behaviour. Interviews were cyclical [30] and developed over the data collection period, initially being open, and then focusing on key mechanisms and testing our emerging theory with different, or sometimes with the same participants, to further develop and test or re-test our emerging CMOC configurations [31]. Initial interviews commenced by asking interviewees how they: (i) were involved in Rounds (facilitator, clinical lead, panellist, audience, steering group member or combination); (ii) understood Rounds to ‘work’; (iii) identified and explained any changes that generated outcomes and; (iv) which aspects of the Rounds process were in their view key mechanisms; (v) and which factors they felt changed the way that Rounds worked (context), which were often accessed through asking participants to reflect on ‘most and least successful Rounds’.

We also held two theory-testing focus groups with Rounds mentors from non-case study sites and key PoCF stakeholders to test and refine our emerging evidence-informed programme theory. There were 9 participants in total; Focus group 1: Mentors/ key PoCF stakeholders (*n* = 4); Focus group 2: Mentors/ PoCF trainers (*n* = 5). The focus group structure included a member of the research team (JM) presenting the nine CMO configurations as if…then causal statements on PowerPoint slides, along with illustrative quotes, and formal theory to inform and stimulate in-depth focus group discussion.

All interviews were conducted in person or by telephone by authors (JM, CT, IM, ER, ML) who are all experienced qualitative researchers. The researchers were motivated to gather participants’ experiences to address the study aims and objectives and researcher details were provided in the participant information sheets. JM knew a small number of participants professionally through previous work, but other researchers had no prior relationships with any of the participants. Interviewees and focus group participants were provided with a participant information sheet and consented in writing to take part in the study. The focus groups were conducted in person in University meeting rooms by authors (JM,CT, ML) and lasted 2 h. Interviews were undertaken in settings chosen by the participants and included confidential spaces such as their own offices or other confidential work spaces. Some interviewees were interviewed more than once to test emerging theory. There were no non-participants present. Interviews and focus groups were audio recorded and transcribed verbatim for analysis. Data transcripts were returned to those interviewees and focus group participants who requested it, for member checking and permission to use extracted quotations.

Interviews and analysis were undertaken concurrently to inform further sampling and further theory refining and testing.

### Data analysis

Our retroductive analysis (going back from below/behind observed patterns or regularities to discover what produces them [32]- see also Fig. 2) moved between inductive and deductive processes, identifying CMOCs and testing researcher ‘hunches’ and the ‘fit’ of explanatory formal theories (see below) and aimed to provide the best possible explanation of how Rounds work for whom and in what circumstances [23]. We identified differing contextual layers which impacted on the activation of mechanisms (this process is not a binary on/off firing, but is akin to a “dimmer switch, where intensity varies in line with an ever evolving context” (page 5) [33]) and used these to explore variation in outcomes to identify the elements of context affecting Rounds implementation [34, 35].

**Fig. 4:**
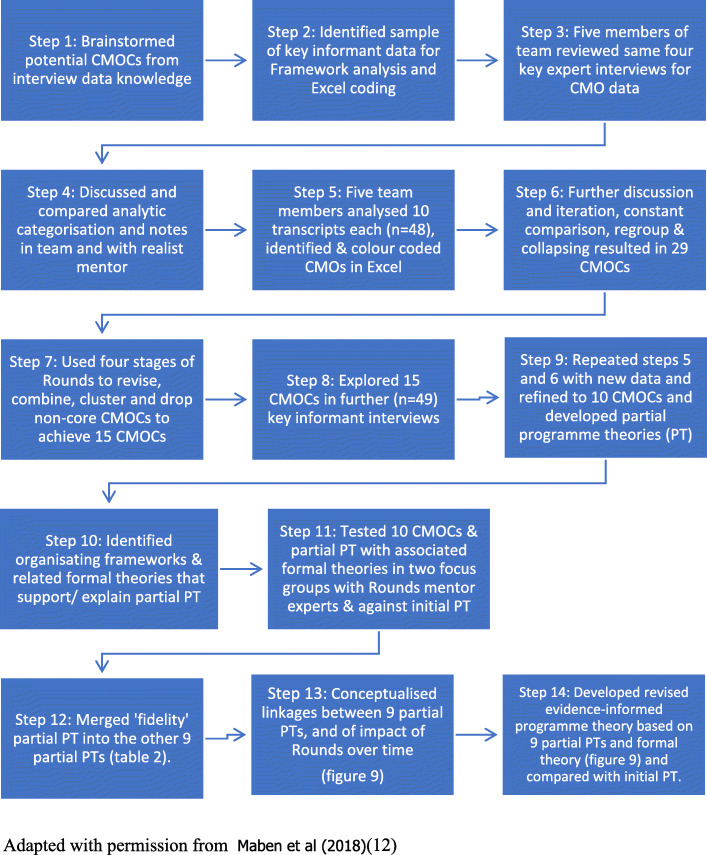
Data analysis process

We approached data analysis in two ways. All interview data were organised in NVivo and Excel using Framework analysis once CMOCs were identified. These data were coded in NVivo 10 to determine and identify participants’ experiences and impacts of Rounds. In organising our data in NVivo 10 we started with and identifying any ‘outcomes’ of Rounds (O in the C + M + O realist analysis process) and worked back to identify causal mechanisms and context. The first step involved creating free nodes (codes) by identifying (reading and highlighting) parts of the data (words or phrases) that related to examples of impact and outcomes resulting from Rounds. Details of the conditions (Context) in which each impact was embedded were extracted, as well as information on how the impact was caused, i.e. produced the the impact and outcome. Second, the free nodes were separated into tree nodes (categories) by deciding which words or phrases had similar meanings and transferring them into an appropriate tree node. Third, the tree nodes were allocated a label that described the theme of the grouped data (e.g. individual, team or organisational level impacts associated with Rounds). Last, the tree nodes were reviewed to select the most dominant themes relevant to the research question. We also analysed the case study key stakeholder interview data (*n* = 48) again with Framework analysis using Excel to determine the C + M + O configurations in these data; Initially five members of the research team (JM, CT, IM, ER, ML), read and coded for CMOCs the same four interview transcripts. The team then met to compare and discuss CMOC notes and analytic categorisation. Then each researcher analysed approximately ten transcripts each (n = 48), identified distinct and overlapping CMOCs, colour coding sections of data that related to context, mechanism and/or outcome. These were then discussed in ten half day meetings supported by a realist methodology expert. The initial 29 CMOCs were further refined and collapsed into 15, 10 and finally 9 CMOCs to reduce overlap and duplication. This was done by testing causative configurations in our repeat interviews with our well-informed ‘experts’ and through further testing in two focus groups with Rounds mentors, and through comparison with the remaining interviews (*n* = 49) (see Fig. 4).

**Fig. 5:**
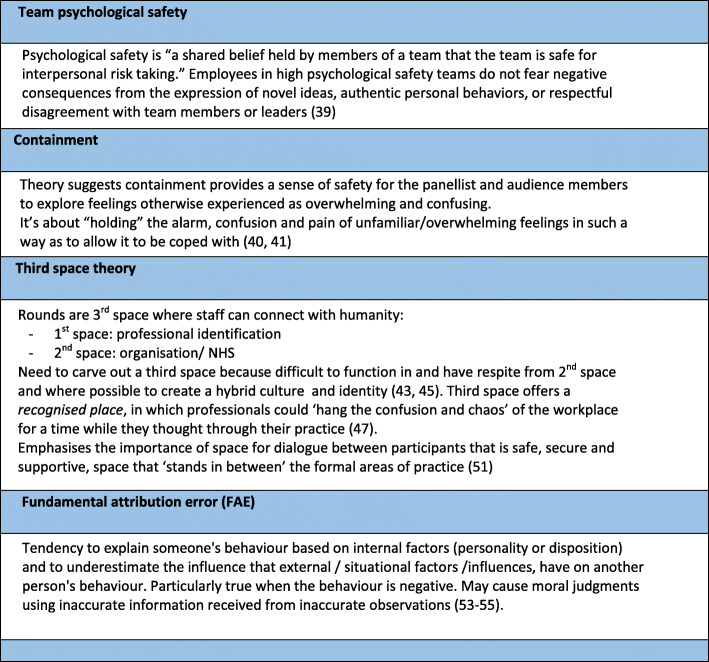
Formal Theories

We also identified a number of formal theories which enabled us to move beyond micro description and link concepts but remain close to our empirical data [36] (page 41). The formal theories we used and draw upon in this paper are presented in Fig. 5 below.

Finally we revisited and refined our initial programme theory in light of our nine partial programme theories with supporting empirical CMOC data, to develop our evidence-informed programme theory (see Fig. 9 below).

We have used pseudonyms to protect the identity of our healthcare organisation sites (rivers for the national sample of 45 sites (49 interviews) and trees for the 9 case study sites (48 interviews) and included interviewees role(s) in Rounds in any quotations used. We have also used Realist Evaluation (RAMESES II) reporting guidelines to ensure rigour in the data analysis and study here [23].

### Findings

As outlined above, we tested and refined our initial programme theory (Fig. 3) resulting in nine interconnected partial evidence-informed programme theories, presented below with their respective CMOCs (see Table 2 below). We compared this data to our initial programme theory and identified the inter-relationships between these nine-partial evidence-informed programme theories to produce our final overall evidence-informed programme theory represented in Fig. 9 (below). We present our findings in three main sections. 1) Case studies and study participants; 2) Partial programme theories and their supporting CMOCs including key aspects of Rounds, where we present three partial programme theories in detail. These three are: (i) Trust, safety and containment; (ii) Counter-cultural/ third space for staff; and (iii) Multiple perspectives enable greater contextualisation of patient/staff behaviours. Finally in section 3) we examine the interrelationships between partial programme theories and present our final programme theory comparing the initial programme theory with our final evidence-informed programme theory.

### Case studies and study participants

From the 45 sites that participated in the Phase 1 mapping study (reported in Maben et al. (2018) [12] nine case study sites were selected to take part in this study. As reported above, these were also purposively sampled nationally (UK) to provide maximum variation (see Table 1 for more details).

**Table 1:**
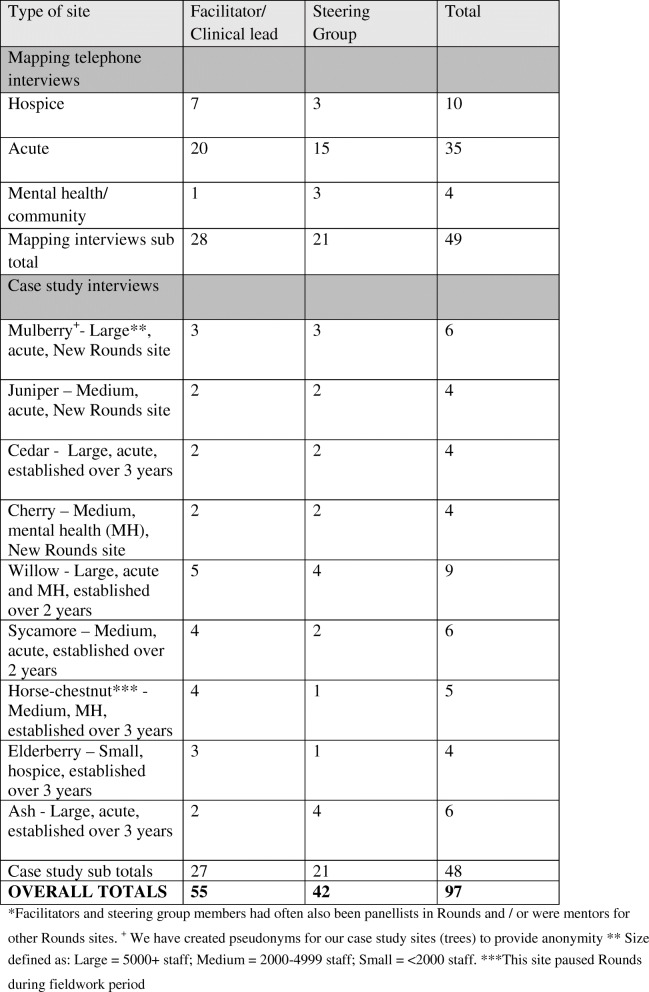
Interview participants* and Case Study sites

In this paper we drew on interviews collected in these case studies with a purposive sample of clinical leads, facilitators, members of steering groups; those who knew most about Rounds (*n* = 48). We also drew on the interviews from Phase 1 national mapping study with 49 facilitators/clinical leads. In total therefore we analysed *n* = 97 key informant interviews for the realist analysis reported in this article.

### Partial programme theories and their supporting contexts, mechanisms and outcome configurations (CMOCs)

The nine partial programme theories are labelled as: Trust safety and containment; Group interaction; Counter-cultural/3rd space for staff; Self-disclosure; Story-telling; Role modelling vulnerability; Contextualising patients and staff; Shining a spotlight on hidden stories and roles; and Reflection and resonance.

In Table 2 (below) supporting CMOCs are provided for each of these theories as ‘If… then’ statements.

**Table 2:**
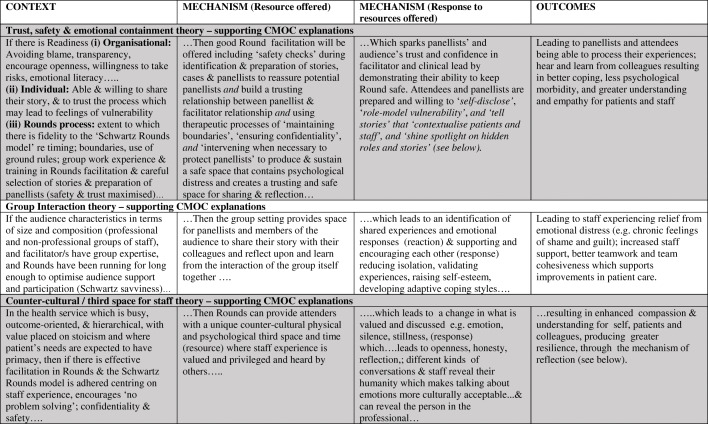

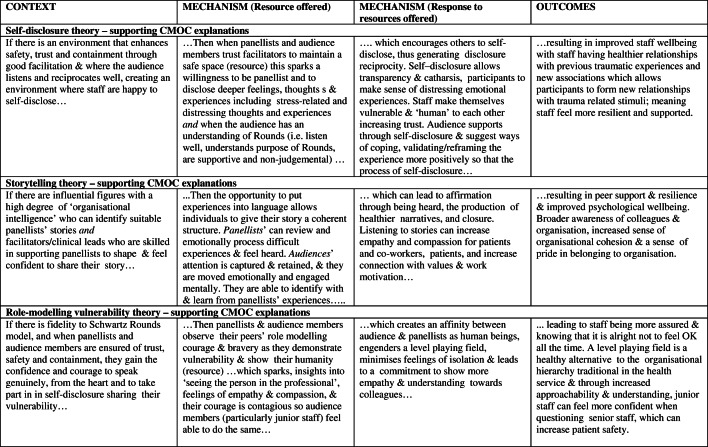

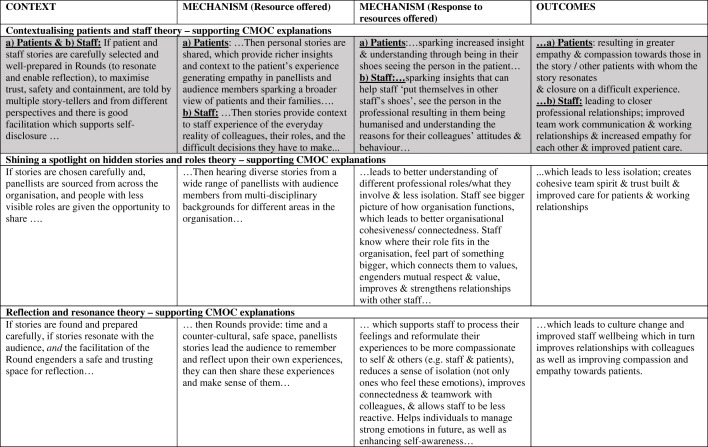
Nine partial programme theories with supporting CMOCs (the three highlighted in grey are presented in more detail below)

Space precludes presentation of all nine partial programme theories (see Maben et al. (2018) [12] for a full description of all nine); so here we have selected three important partial programme theories to present with illustrative data, drawing on formal theory to further explain how Rounds work to produce their outcomes, and to strengthen the transferability of our findings.

### Key aspects of rounds: three partial programme theories in detail

These three partial programme theories were selected for more in-depth presentation as they represent important and key aspects of Rounds at different stages: (i) trust safety and containment is an important pre-requisite enabling Rounds to work, (ii) counter-cultural /3rd space for staff represents the culture of the Round itself; and (iii) exemplifies how the telling of stories in Rounds results in an increased contextualisation of patients and staff to generate impact and outcomes.

#### Trust, safety and containment

**Fig. 6:**
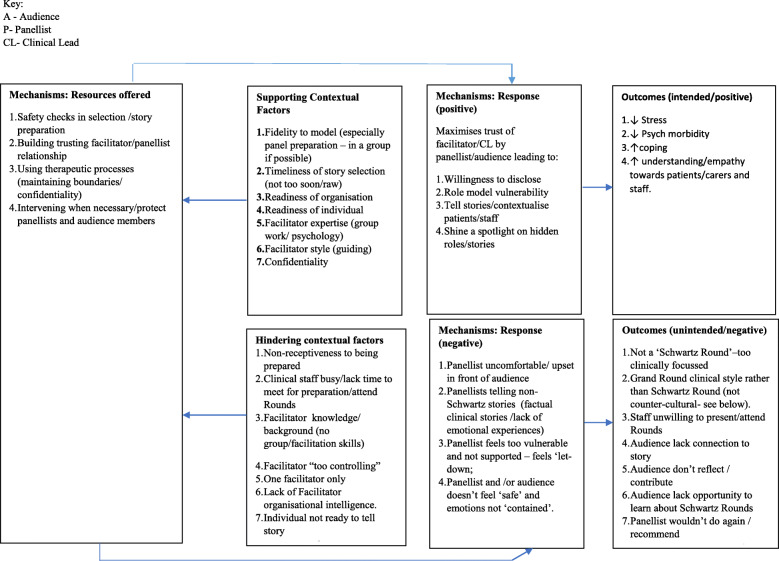
Trust, Safety and Containment: specific contextual factors that hinder or enable the mechanisms to fire

Our data suggests that key to the successful implementation of Rounds is trust, psychological safety and emotional containment. Mechanisms allowing Rounds to feel safe, trusting and which draw upon the psychotherapeutic notion of ‘containment’, refers to the atmosphere the facilitator creates that conveys a sense of safety to enable people to focus upon their emotions. We observed examples of how this is achieved by facilitators during their panel preparation work with Rounds presenters (Mechanism [resource]). For example, helping presenters reflect on the consequences of sharing their study with a wider audience, and how they might do this safely (Mechanism [response]) building a trusting facilitator/panellist relationship and with the audience (Outcome). This enabled everyone to develop confidence in the facilitator’s use of other therapeutic processes (maintaining boundaries/confidentiality) and their willingness to step in if needed to protect the panellists and audience contributors from judgemental or inappropriate comments, and had faith in the Schwartz Rounds process itself (Outcome). In response to the resources offered by Rounds, certain contexts can support reasoning to activate mechanisms to ‘fire’ optimally on a continuum where intensity varies in line with changing context (see Fig. 6 below). These contextual factors include: fidelity to the model (see Leamy et al. (2019) [28]; safe and good panel preparation (where staff pre-prepare and shape the stories they will share in Rounds with facilitators), in a group if possible (see McCarthy et al. (2020) [37]); the timeliness of story selection (not too raw or new); the readiness of the organisation and individuals; and facilitator expertise (group work/ psychology) and style (guiding) (see Fig. 6).

Key aspects of safety noted by our interviewees included pre-Round safety checks (Mechanism [resource]), such as checking panellists understand the potential consequences of publicly telling their story and countering the ‘urge to confess’. Conversely an ‘unsafe’ Round would include panellists and audience members experiencing embarrassment, rejection, reprisal or blame, or experience bullying and punishment for speaking out (Outcome and subsequent Context). Thus, in Rounds where safety may have been compromised, participants’ feelings of trust, safety and containment can be damaged (Mechanism [response]) affecting their willingness to attend, participate, and disclose experiences (Outcomes) (see Fig. 6).

Rounds stories often meant supporting staff revealing their vulnerability and potential shame. These were all explored where possible in panel preparation prior to the Round, for example panellists may say:

*‘I’d really like to tell this but I'm quite embarrassed about that and I'm not sure I could tell’, so that might be about making a mistake (…) so I always check about safety, about whether they feel safe enough to tell it.* (Cherry-1-Facilitator).

Timing of stories was important (Context), and some stories were deemed ‘too soon’ or ‘too raw’ to tell by facilitators and so were held for later Rounds.

*It was quite a traumatic case [pregnant girl diagnosed with cancer] (…) at the time it was just too raw to be done, when I had my meeting with [facilitator] I found it quite upsetting and said ‘actually I don't think it’s appropriate, a lot of people who come are actually still looking after her (…) I think in a way that worked very well having the interviews before the Schwartz Round because (…) the timing of it just wouldn't have been correct*. (Cedar-26-Panellist).

In Rounds themselves, attendees noted the importance of good facilitation (Mechanism [resource]) *“I think I’ve always been quite impressed with the way that space is held”* (Elderberry-13-Panellist and frequent attender). Many panellists and attenders we interviewed suggested that facilitator skills were key to a successful Round: *I’ve been really impressed (...) the facilitators have managed to keep it to time, keep it controlled, but yet without you really being aware that they’re doing that at all (*Elderberry-10-Panellist and frequent attender).

Safety and trust were identified by facilitators as cumulative, influencing whether people shared and which stories are offered. Participants’ feelings of trust, safety and containment (Outcomes) develop through repeated exposure to safe Rounds (Mechanism [resource]) and repeating of the ground rules (Mechanism [resource]):

*It’s how you set it up at the beginning, providing, setting the ground rules. (Willow-1-Facilitator).* Safety and trust (Context) are essential for staff to share taboo subjects such as shame (Outcome): *issues of feeling shame (…) you don’t necessarily feel comfortable in sharing with your colleagues (..)(shame) is almost brought to light and almost solved.* (Horsechestnut-01-Administrator-and-Rounds-Attender). One facilitator spoke of not over-reacting to emotions, an important aspect of containment:

*I think mostly it’s implicit around communicating and being in a way that is respectful, that you trust that people are grown-ups here, that they can express emotions and they will be fine. So not over reacting to emotional expression. Being with it and accepting it and you know sometimes saying look around, you know, people too are feeling that way here in the room so its universal (Willow-1-Facilitator).*

The process of sharing in a safe and contained space (Context) meant that attenders learn to cope with damaging, overwhelming, or potentially explosive emotions [38]. Our data suggest that the safe space (Mechanism [resource]) allows vulnerability (Mechanism [response]), an important first step towards trust (Outcome): *it allows you just to be vulnerable, to share about a time or an experience in your life and I just think that really just maybe energises others to feel safe (…) it just pulls down so many barriers. (Elderberry-11-Panellist-&-Steering-Group-Member).*

Psychological safety ‘describes a team climate characterised by interpersonal trust and mutual respect in which people are comfortable being themselves’ (p354) [39] which our data supported, for example: *Schwartz Rounds brings people together from all walks of life, all jobs and it gives them permission to speak and it feels safe. (Elderberry-08-Stakeholder)*.

Furthermore, the formal theory of psychological safety suggests containment (Mechanism [resource]) provides a sense of safety for the panellist and audience members (Mechanism [response]) to explore feelings that may otherwise be experienced as overwhelming and confusing (Outcome) by “holding” the alarm, confusion and pain of unfamiliar or overwhelming feelings (Mechanism [resource]) allowing it to be coped with (Outcome) [40]. The facilitator needs to manage their own sense of uncertainty (Context) to provide confidence to enable panellists and Rounds attenders to believe their difficult feelings can be managed, explored and understood (Mechanism [response]) allowing unsatisfactory coping mechanisms to be avoided (Outcome) [41]. Confidentiality is crucial if members are to take risks necessary for real growth [42]. Our data provided evidence to support these formal theories:

*It’s set up to be a safe, confidential forum (…) a place for people to express their feelings, some ‘containment’ both from the panel and other people in the audience, (…) for it to be OK to express feelings that might seem strange, unacceptable, awful.* (*Anonymised-site-17-Panellist-speaking-from perspective-of-facilitator/psychiatrist).*

#### Counter-cultural/ third space for staff

The counter-cultural and ‘third space’ theory of Rounds was not explicit in our initial programme theory, but recent literature and analysis of our data shifted our thinking. In 2016, Barbara Wren wrote: *“the process of Schwartz Round implementation is in many ways counter-cultural. Good Rounds shift an organisation and its workers away from their default position of urgent action, reaction and problem solving to an hour of stillness and slowness*” [13].(p41). Our data supported this and helped us identify Rounds as a third/counter cultural space (see Table 2). Rounds allow staff a break away from the normal hectic, fast-paced, target and protocol driven, hierarchical culture of healthcare work (Context). In doing so they enter a confidential, safe space where stillness, silence and reflection is valued (Mechanism [resource]) allowing staff to be more present and sit with discomforting emotions (Mechanism [response]). This facilitates greater ease in their often-difficult work with patients, increasing job satisfaction and buffering negative aspects of healthcare staff experiences (Outcomes). Staff spoke of *‘leaving things at the door’* and being in a different ‘zone’ to reflect allowing staff the opportunity to consider doing things differently:

*(Rounds provide) uncluttered headspace (…) where you can put the brakes on, you can stop, you can leave that constant drive for performance targets at the door and have that clarity where you can just breathe and start thinking with like-minded colleagues who experience the same pressures, (..) being able to sit with the emotion, be with the emotion, that you normally just press, push it down, push it down, push it down, move to the next task, (..) so, that ability to reflect on your practice and have that headspace (…) that’s where that opportunity to do things differently comes from. (Horse-chestnut-07-Clinical-Lead).*

Over time, participants reported that they came to appreciate the different space provided by Rounds and realised they could take action. However, it was not a requirement and that they were not forced to act or take a position, they could just listen and reflect internally. The silence and stillness as particularly valued by some:

*I think it’s [silence] a whole new concept for most people in the audience and sometimes you can literally see people sort of relax into that, ‘oh it’s ok then, she’s given us permission’ you know […] so to be sort of told the silence is ok just go with it may be a wonderful release. (Mulberry-367-Facilitator)*

*Because the good thing about the Round, particularly the fact that you can attend and not speak (..) is that you could have a moment of stillness where you just observe complexity but you don’t have to do anything with it, because clinicians are working with complexity all the time and are forced to take a position. (Focus group, 1).*

**Fig. 7:**
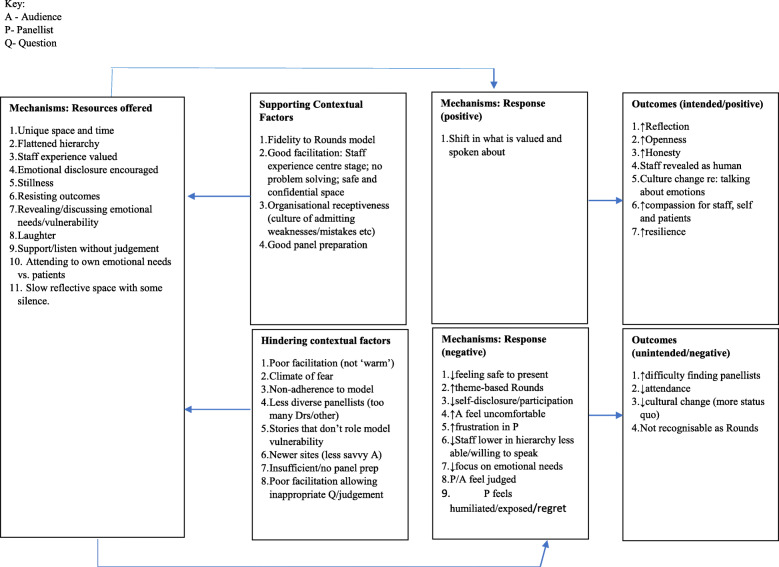
Counter-cultural third space: specific contextual factors that hinder or enable the mechanisms to fire

Again we identified that certain contexts supported the activation of mechanisms to ‘fire’ optimally so that there can be a shift in what is valued and spoken about (see Fig. 6 below). These contextual factors include: fidelity to the Rounds model (see Leamy et al. (2019) [28]); Good facilitation and panel preparation; where staff experience is centre stage; where there is no problem solving and safe and confidential space (see McCarthy et al. 2020 [37]); as well as organisational receptiveness (culture of admitting weaknesses/mistakes etc) (contexts) (see Fig. 7).

We iteratively tested our findings regarding Rounds being a counter-cultural space in relation to ‘Third space’ formal theory. First and second spaces are two different spatial groupings where people interact physically and socially [43–45]. Our data suggest Rounds are ‘third spaces’ (Mechanism [resource]) where staff can (re-)connect with their values and humanity (Mechanism [response]) with first and second spaces potentially being 1) professional/role identification and values (everyday work) and 2) NHS-healthcare/organisation spaces (imposed work practices) [46]. Staff spoke of Rounds as spaces where hierarchies were flattened and everyone was connected by the same struggles and their humanity (Outcomes):

*I think there’s also something about flattening hierarchies, so we've had the most senior manager in our locality on a panel with a receptionist and with, I think, a nurse. Seeing them all side by side and saying very similar things and clearly struggling with similar dilemmas brings everybody down to the same human level, which is helpful. (Taff-138-Clinical lead).*

Third space formal theory offered four applications for our work on Rounds including: (i) recognised space; (ii) community space; (iii) conversational space; and (iv) learning space. A *recognised space* offered a place where professionals could ‘hang the confusion and chaos’ of the workplace for a time while they thought through their practice [47]. A community space was not centred around targets and outcomes but provided a community of commitment [48] and creation [49] relating to ‘what people care about and want’ to create together [50]. In terms of a *conversational space*, this acts as a space for dialogue between participants that is safe, secure and supportive, that ‘stands in between’ the formal areas of practice [51]; (p43–44). Our data supported this, for example:

*I understand Schwartz Rounds as a place or a forum to just kind of offload things, in a shared sense. In a safe place, (..) it’s a place of reflection, space, thinking, creativity, challenge sometimes because some of the things that people share touch on your own life. (Elderberry-01-Panellist- frequent attender-steering group-member).*

A *Learning space* offers, “places of transition, and sometimes transformation, where the individual experiences some kind of (..) a shift in identity or role perception so that issues and concerns are seen and heard in new and different ways” [52](p8–9). Interviewees spoke of Rounds as a reflective learning space which impacts on patients’ experiences:

*I think one of the biggest impacts is (…) on the quality of people, patient’s experience (..) that space to reflect in a safe space away from the coal face of what is happening, surely has an impact on how good a practitioner someone is (…) it gives them a little bit of space to reflect in a way which is well structured. (..) helping people really gather up and pause and think about actually that tacit knowledge which you don’t get from anywhere else other than those conversations. (Juniper-26-Steering-Group-Member).*

Reported outcomes included improved compassion and understanding for others (including patients and staff) and self, creating greater resilience, via the mechanism of reflection (Outcomes) (see Table 2). An example was given by a facilitator: *The reflection that we do as a team on our practice (..) just helping me to be better at being compassionate and understanding and open to people recognising the impact on ourselves emotionally. And just that greater resilience. (Ash-05-Facilitator).*

Others suggested there had been wider cultural changes (‘ripple’ effects) through the counter-cultural third space offered by Rounds (Mechanism: resource): *It’s sort of softened the ground for things to grow, the kind of conversations (…) are very different to the ones (..) about a year ago. (Carmel-385-Facilitator).*

#### Multiple perspectives enable greater contextualisation of patient/staff behaviours

This was also not in our initial programme theory, but this refined partial programme theory and supporting CMOCs further illuminates how Rounds work as staff support and teach each other (Mechanism [resource]) how to better connect with patients (and each other) (Outcomes). Panellists were willing to self-disclose, show their vulnerability and tell their story publicly (Mechanism [response]). In doing so, audience members and fellow panellists gain an expanded knowledge of patients and staff (Outcome). They hear multiple perspectives about the same patient from different colleagues and hear more about the external and contextual factors which impinge upon individual patient’s and staff’s decision-making and behaviour (Mechanism [resource]). This helps people appreciate what other colleagues are going through (Mechanism [response]) and show greater kindness and compassion towards them (Outcome):

*I think it makes me more sort of appreciative day to day about what other people face in their jobs. I think it makes me more likely to act with kindness and a bit more compassion for my colleagues (…) seeing people participate and share comments about their personal experiences has altered the way I think about some people. And I've been able to see more of them as a person (…) (and) feel more positive about them generally. (Elderberry-13-Panellist-and-frequent-attender).*

Staff also reported gaining a longitudinal rather than cross-sectional perspective (Mechanism [resource]) of what happened to a patient or colleague. In short, they are able to put themselves in the patient’s shoes or get a glimpse into the everyday world of that individual staff member (Outcome) (see Table 2).

Drawing on formal theory, Fundamental Attribution Error (FAE) enhances our understanding of our data. FAE is the attribution of internal factors (e.g. personality) to make sense of another’s behaviour, without full consideration of the impact of situational and external elements on that behavior [53–55]. In Table 2 we present patient (a) and staff (b) data which suggests that FAE applies to the contextualizing of both of these groups. Rounds stories which revealed staff vulnerability (Context) were reported to be particularly moving (Mechanism [response]), as they gave audience members an appreciation of the pressures colleagues may be under (Mechanisms [resource]). This led to greater empathy, tolerance, and generosity (to self and others), which in turn led to more trust, compassion and kindness to each other (Outcomes). During a Round, people are reminded that the behaviour of a patient or colleague is shaped by contextual factors and are given the opportunity to simultaneously account for both situational and behavioural awareness in order to make sense of the patient’s, carer’s or staff member’s behaviour:

*I think I’ve got a bigger understanding as to how other people’s minds work. I have a bigger understanding as to what other people have to put up with in their jobs, how challenging they are […] really understanding where other people are coming from (and I have) more empathy towards other staff members and colleagues, but also patients as well. (Willow-178-Clinical lead).*

Listening to stories at Rounds (Mechanism [resource]) also gave the audience insight into the everyday lives of patients (Mechanism [resource]) and a greater appreciation for why people behaved in certain ways (Mechanism [response]), shifting their understanding (Outcome):

*Well it’s certainly changed the way I view patients (..) I think I’m much more aware of their back story, if that makes sense. So for example some of the Rounds [included] occupational therapists, they’ve talked about seeing the patient’s home life, they’ve […] visited their house and how chaotic and dishevelled many people’s lives are outside the trust (Looe-381-Facilitator).*

This understanding (Mechanism [response]) gave audience members the space to reflect upon difficult cases (Mechanism [resource]), which in turn led to greater empathy and an appreciation of the complexity of care work carried out day to day (Outcomes):

*It’s just about having a broader view or whether it’s about reconnecting to their values (…) obviously they’re exposed to patients, they hear stories every day, so it’s the way in which they’ve heard this story that’s changed their empathy somehow (…) there’s two things happening. One is maybe a sense of closure for those who were involved in the case, but the other thing is the broader view of patients and the empathy is not just the patient you’re hearing about? (Focus group-8).*

**Fig. 8:**
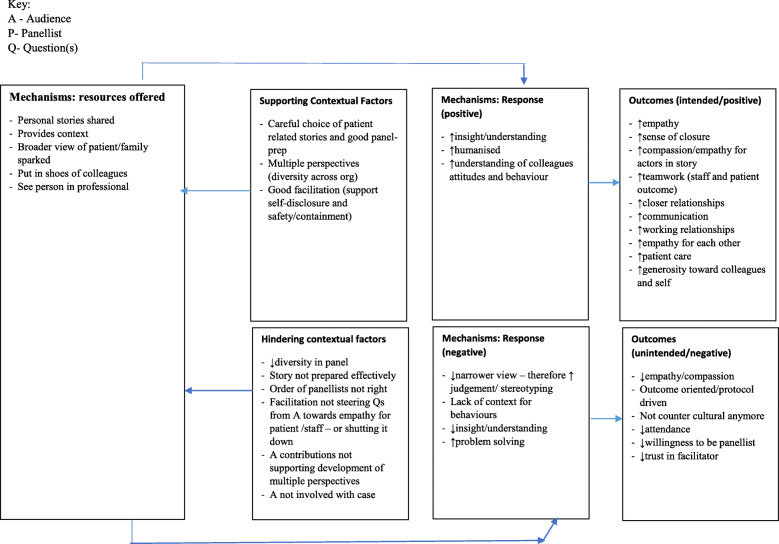
Contextualising patients and staff: specific contextual factors that hinder or enable the mechanisms to fire

The mechanisms supporting these outcomes include the telling of stories (Mechanism [resource]) that allow personal and more contextualised insights into problems shared and heard (Mechanism [response]) enabling staff to be put in shoes of patients and/or colleagues, seeing the person in the patient or professional (Outcomes) (see Fig. 8). Favourable contextual factors that allow these mechanisms of rounds to activate optimally include: careful choice of patient related stories and contextualised staff stories in good panel-preparation; multiple perspectives (reflecting the diversity across the organisation or within a patient case); and good facilitation, which supports self-disclosure and safety and containment (see Fig. 8). Conversely a Round where the context would hinder the firing of the mechanisms in this CMOC would include having fewer in number or less diversity in panellists; if the stor(ies) are not prepared effectively to show context; and if audience and panellists’ contributions do not support the development of multiple perspectives (Contexts) (see Fig. 8).

### Interrelationships between partial programme theories and final programme theory

**Fig. 9:**
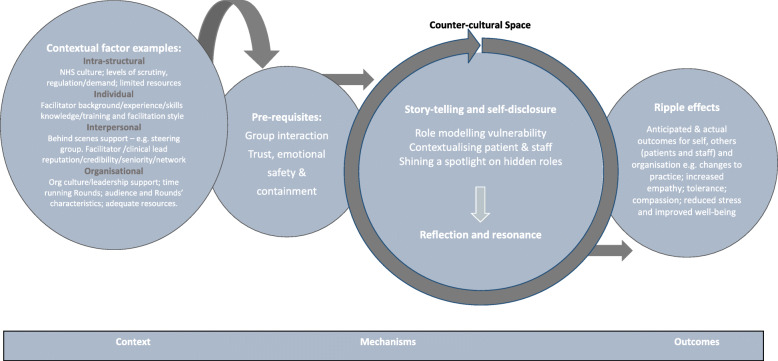
Final, evidence-informed programme theory illustrating how programme theories supported by CMOCs link together and model to explain how Rounds ‘work’

The inter-relationships between each of these nine partial programme theories was examined and tested with reference to context to identify causal outcomes which resulted in our final evidence-informed programme theory (Fig. 9). This final evidenced -informed programme theory (Fig. 9) provides additional insights into how Rounds work to generate their effects.

Figure 9 shows how the four layers of contextual factors on the left of the diagram impact on the firing of the mechanisms in the middle; both the two pre-requisites (group interaction and trust, emotional safety and containment) as well as those that work in the Rounds itself (storytelling and self-disclosure; role modelling vulnerability; contextualising patients and staff, and shining a spotlight on hidden roles which leads to reflection and resonance to provide a counter-cultural space), which produces outcomes identified on the right of the Fig. 9 diagram. Drawing on formal theories, we propose that *Rounds provide a counter-cultural / third space for staff in which, resonance, reflection, containment and trust as well as safe and non-judgemental space are significant features.* Reflection and resonance can be triggered by hearing stories and witnessing the self-disclosure of panellists and audience members. *Storytelling is central to Rounds and panel preparation is crucial for crafting and telling powerful emotional stories of staff experiences and for maintaining emotional safety. Rounds offer the resources (*as part of the process of self-disclosure and story-telling) to support reasoning and response that contextualises patients and staff; role model vulnerability; and ‘shines a spotlight on hidden roles’ to staff attending Rounds. *However, o*ur data revealed that a number of contextual factors were necessary to enable Rounds mechanisms to ‘fire’ optimally by allowing attendees and panellists to feel confident to speak in a group; allowing themselves to be vulnerable and to share their stories with emotional safety; and containment, facilitating trust and open and honest dialogue. As identified in Figs. 6, 7 and 8 above, hindering contextual factors which contribute to Rounds being less successful can relate to different contextual layers, including the characteristics of the facilitator or audience, preparation for the Round, and organisational factors. Organisational contexts of the Rounds provider included where there is a climate of fear, non-adherence or lack of fidelity to the Schwartz Rounds model, or when sites are new to running Schwartz Rounds, so audience and panellists are less ‘Schwartz Rounds savvy’. Hindering factors in Rounds preparation included a lack of diversity in panellists, or panellists having been insufficiently prepared (e.g. non-receptiveness to being prepared, clinical staff busy/lack time to meet for preparation/ attend Rounds). The selection of panellists and stories is an important contextual factor, for instance, where stories that don’t role model vulnerability, have not been prepared effectively or an individual panellist is not ready to tell their story. Rounds facilitation is optimal where there are two facilitators who can share the task between them. Rounds are less successful when facilitators have limited group/facilitation skills or organisational intelligence, where their communication style is not conducive to psychological safety, e.g. “too controlling”, “lack of warmth”, or they facilitate the audience contributions inadequately, for instance where audience contributions do not support the development of multiple perspectives, or facilitators allow inappropriate questions or judgements of the panellists, which can impact on psychological safety.

In terms of generating outcomes, it was sometimes difficult for first-time Rounds attenders to understand that solving problems is not the primary aim of Rounds and some attenders felt frustrated at first that Rounds did not lead to identifiable outcomes. However interviewees described how, with experience, staff began to value the unique space created by Rounds and understood that they *could* make changes as a consequence of their insights from Rounds, but it was not a requirement. Interviewees described behavioural modifications and identified some instances of ripple effects discerned in everyday practise across the organisation. Some examples of these include protocol changes, changes to culture and conversations, and support groups established for specific staff.

## Discussion

This is the first evaluation of Schwartz Rounds to describe, through robust application of Realist methodology, how and why Schwartz Rounds work to produce outcomes previously undocumented in original descriptions and subsequent evaluations of the intervention [9, 10, 15, 16, 18, 20, 56–64]. It is also the first to make visible the implicit programme architecture underpinning the design of Rounds, and to identify the four stages of Rounds.

Our evaluation resulted in a final evidence-informed programme theory – synthesising 9 inter-linked partial programme theories– to describe how Rounds ‘work’ to produce their outcomes. Our data identified that there is less explicit ‘teaching’ in Rounds in the UK than our initial programme theory implied, but supported our initial programme theory that Rounds reportedly strengthen empathy, compassion and relationships between staff as well as strengthening the patient-caregiver relationship. These outcomes of Rounds have been reported in previous evaluations of Rounds [27], but previous studies had not systematically explored how or why the outcomes occurred, nor the impact of different contextual factors on them occurring. More recently, a mixed methods evaluation of Rounds in mental health services [65] reported benefits of attending Rounds as a) the ability to express emotions and b) sharing similar emotions and experiences to colleagues and feeling empathy and recognition with colleagues’ experiences. These findings fit with our evidence-based programme theory, though our findings go beyond this to explain how and why Rounds provide an environment where these benefits can be realised (and indeed to explain when they don’t).

Our evaluation suggests that when run as intended with fidelity to the UK Schwartz Rounds model [28] and in optimal contexts, Rounds provide staff with a valuable space to speak together in an open and honest way about the realities of their work. This is a unique space for healthcare staff, with no other known interventions offering such a space for all staff to come together [27]. The impact of Rounds can take time to develop but the benefits are many including: greater compassion and empathy between co-workers and towards patients; mutual support amongst staff; more effective communication and teamwork; decreased feelings of isolation; and changes to practice, including changes to culture and conversations, protocol changes, and specific staff support groups established. This has implications for both implementation of Rounds and their evaluation. This ‘cumulative’ impact (or time-effect) is a common feature of complex interventions. Attempts to understand contextual variability when such interventions are implemented in real life led to the development of Normalisation Process Theory [66, 67]. This theory purports that implementing is not the same as integrating an intervention into everyday practice [67], the latter requiring coherence (or sense-making); cognitive participation (or engagement); collective action; and reflexive monitoring (formally and informally) [68]. Our research supports the impact of these constructs on how workable and embedded Rounds were across our case study sites.

In Realist evaluation, Pawson suggests, for social interventions such as Schwartz Rounds, the mechanisms are the cognitive or affective responses of participants to the resources offered [35] and these lead to intermediate and longer term outcomes which are also cognitive or affective responses. Thus these cognitive and affective responses and the ‘altered state of attendee’ is the central outcome of all our supporting CMOCs reported here. We have detailed these altered states and outcomes which include: enhanced feelings of empathy and compassion for one’s self, co-workers, patients and their relatives; improved resilience; greater honesty and openness; and organisational cultural change.

It should be noted that Rounds were not for everyone and did not ‘work’ for all. Staff wellbeing strategies require a multi-pronged approach, incorporating interventions or approaches that support wellbeing from prevention to treatment of ill-health, and that target individuals, teams and whole organisations [27, 69]. Organisations therefore may consider implementing Rounds alongside other interventions.

A unique feature of this study is the focus not only on how Rounds work, but also when they don’t work (or do not work as effectively). This is a major strength of the methodology we have used, ‘untangling the complexity of real-life’, enabling learning from where Rounds do not work so well to produce optimal outcomes as well as where they did [70]. We have detailed potential hindering contextual factors and responses which may result in negative or intermediate outcomes [37], or that may dim or brighten the mechanisms including: difficulty identifying new stories (dim); staff feeling supported to take risks and disclose (brighten); and lack of organisational support for Rounds and new facilitators taking on risky stories (dim). Our data support the idea that there is a continuum effect for some mechanisms, which, rather than ‘firing’ or ‘not firing’, operate on a ‘dimmer switch’ [33], with various contextual factors turning up the intensity in relation to an ever-evolving context.

### Strengths and limitations

One of the main strengths of our study is that it is the first to use a realist evaluation to understand the complex processes at play in Rounds. Rounds were a challenging intervention to evaluate. Much of what was happening in the room during Rounds was on a cognitive level (in people’s heads) and so not visible or always reported, not least because not everyone spoke in Rounds. To counter this we tried hard to speak to people immediately after Rounds where possible and asked them to recount their feelings and experiences of the Round that we had just observed (so we had a lot of immediate contextual knowledge). We looked long and hard to find those who could tell us when Rounds were both ‘successful’ and ‘less successful’. We also observed Rounds (which whilst not reported here – see Maben et al. (2018) [12] for more details, but these observations informed our knowledge, understanding and theorising of Rounds considerably). Previous evaluations of Schwartz Rounds have focused primarily on the impact of Rounds on audience members. We undertook interviews with a range of staff, including varied professional groups and support staff; those who had been panellists, facilitators and steering group members; regular and infrequent attenders; and also those who might have stopped attending. Furthermore, we used RAMESES II guidelines for conducting and reporting our study. Purposive sampling was adopted which allowed for variation across the case study sites in terms of context and intensity of data collection (i.e. number, depth and breadth of interviews undertaken). Our evidence-informed programme theory and use of formal theories and our work identifying the components of Rounds including the four stages adds considerably to the evidence base on Rounds and has examined how, why and for whom Rounds had an impact.

In terms of limitations, our outcome data is self-reported data. Our case study observations did not include observation of mentor**-**facilitator/clinical lead debriefing and support meetings, providing an opportunity for facilitators/clinical leads to reflect upon where Rounds sit within the wider organisational context, which could have provided further data on contextual variations between sites. It was not possible to observe ripple effects of Rounds (actual changes which occurred as the result of attending Rounds) because interviewees found it very hard to identify concrete examples of changes in practice, and whilst we had originally planned to try and observe these in practice, in reality, this proved impossible. Finally, our study only focused on staff experiences, not patient experiences. We did consider including the impact of Rounds on patient experience, but the difficulties of attribution of Rounds effects of Rounds on patients (confounders) meant this was not possible. However, substantial evidence now links staff wellbeing with patient experience of care [71], justifying the focus on staff wellbeing.

Organisations already running Rounds or keen to do so can learn much from our in-depth work (which we have distilled into a film and guide to implementation and sustainability for organisations [72, 73]. In particular, the identification of both the supporting and hindering contextual features reported here that support the mechanisms of Rounds to fire optimally or be dimmed, which can help organisations to understand why Rounds’ impact may vary over time and between Rounds.

## Conclusion

Working in a healthcare system marked by rising complexity, staff shortages, increased scrutiny and limited resources can take an emotional toll on staff. We have identified supporting and hindering contextual factors, and where supportive contexts exist with high fidelity to the model [28], Rounds provide staff with a safe and reflective space to share stories, amongst their peers, about the emotional, ethical and social impact of their work. In this case, Rounds attendance was associated with enhanced feelings of empathy and compassion towards patients and staff, as well as positive changes in practice, potentially supporting organisations to improve the quality of patient care and organisational culture. This paper offers a framework, based on causal explanations and evidence-informed programme theory, which outlines how Rounds work, for whom and why, that can support their optimal implementation and also support any future evaluation of Rounds. Our evidence suggests that Rounds are likely to work best in conjunction with other interventions (as they are not accessible or right for everyone) and may be best placed as part of a wider organisational improvement strategy due to the unique resources they provide. In particular, Rounds as an organisation-wide intervention, can provide a counter-cultural space for open dialogue and reflection, crucial for the delivery of high-quality patient care and can support healthcare staff to do the very challenging roles society asks of them.

## Data Availability

The datasets generated and/or analysed during the current study are not publicly available due to confidentiality but are available from the corresponding author on reasonable request.

## Abbreviations

A: Audience
CMOC: Context, Mechanism, Outcome Configurations
IPT: Initial Programme Theory
NHS: National Health Service
NPT: Normalisation Process Theory
P: Panellist
PoCF: Point of Care Foundation
Q: Question
RAMESES: Realist And Meta-narrative Evidence Syntheses: Evolving Standards
SCCC: Schwartz Center for Compassionate Care
UK: United Kingdom
